# Decorating bacteria with self-assembled synthetic receptors

**DOI:** 10.1038/s41467-020-14336-7

**Published:** 2020-03-10

**Authors:** Naama Lahav-Mankovski, Pragati Kishore Prasad, Noa Oppenheimer-Low, Gal Raviv, Tali Dadosh, Tamar Unger, Tomer Meir Salame, Leila Motiei, David Margulies

**Affiliations:** 1 Department of Organic Chemistry, Weizmann Institute of Science, 7610001 Rehovot, Israel; 2 Chemical Research Support, Weizmann Institute of Science, 7610001 Rehovot, Israel; 3 Life Sciences Core Facilities, Weizmann Institute of Science, 7610001 Rehovot, Israel

**Keywords:** Synthetic biology, Fluorescent probes, Supramolecular chemistry, Self-assembly

## Abstract

The responses of cells to their surroundings are mediated by the binding of cell surface proteins (CSPs) to extracellular signals. Such processes are regulated via dynamic changes in the structure, composition, and expression levels of CSPs. In this study, we demonstrate the possibility of decorating bacteria with artificial, self-assembled receptors that imitate the dynamic features of CSPs. We show that the local concentration of these receptors on the bacterial membrane and their structure can be reversibly controlled using suitable chemical signals, in a way that resembles changes that occur with CSP expression levels or posttranslational modifications (PTMs), respectively. We also show that these modifications can endow the bacteria with programmable properties, akin to the way CSP responses can induce cellular functions. By programming the bacteria to glow, adhere to surfaces, or interact with proteins or mammalian cells, we demonstrate the potential to tailor such biomimetic systems for specific applications.

## Abstract (web summary)

Cell surface proteins mediate the interactions between cells and their extracellular environment. Here the authors design synthetic biomemetic receptor-like sensors that facilitate programmable interactions between bacteria and their target.

## Introduction

Various cellular processes are mediated by the binding of cell surface proteins (CSPs), generally membrane receptors, to various extracellular signals (e.g., ions, small molecules, proteins, or cells) in their surroundings^1^. These interactions, which allosterically change CSP structures, can mediate physical processes such as adhesion or transport. In addition, the CSP–ligand interactions can trigger subsequent cell signal cascades that eventually alter the cell’s behavior. Bacterial adhesins, for example, can mediate non-specific adhesion to solid supports^2^. In addition, these CSPs can selectively interact with host cells and trigger the expression of virulence genes that provide the bacteria with pathogenic properties^2,3^. Another example of the way membrane proteins’ responses can induce cellular functions is the binding of quorum sensing (QS) receptors to small molecules^4^ or peptide autoinducers (AIs)^5^. Here, QS receptor activation by AIs induces a collective gene expression behavior that can ultimately result in the formation of biofilm or bioluminescence. Similarly, binding of receptor tyrosine kinases (RTKs) to their ligands initiates cell growth and differentiation^6^. To enable the cells to dynamically adapt to changes in their environments, CSP functions are tightly regulated. Regulation is achieved owing to the reversibility of the CSP–ligand interactions, which makes them dependent on the concentrations of the external cues^4–6^. In addition, the cells’ response to their environment is controlled by feedback loops that dynamically alter the CSP structure, local concentration, or composition^7–9^. For instance, following host infection, adhesins undergo posttranslational modifications (PTMs) that can disrupt inter-bacterial adhesion^3^. Similarly, activation of bacterial QS receptors by AIs^10^ or growth factor receptors by their ligands^9^ can differentially change the receptors’ expression levels. These examples^2–10^ thus highlight the complex interplay between CSPs and external molecular signals. The latter reversibly interact with CSPs, change their conformation, and subsequently trigger cell signal cascades that can further alter the CSP structure, composition, and expression levels.

In recent years, considerable attention has been devoted to developing protein binders based on oligodeoxynucleotide (ODN)–small-molecule conjugates^11–27^ that, similar to CSPs, can respond to external stimuli and undergo dynamic structural changes that enable them to reversibly interact with proteins and regulate their functions^11–21^. We have recently developed protein binders based on ODN–small-molecule conjugates^21–23^ and have shown that such binders can be designed to reversibly interact with different proteins^21,22^ and even mediate unnatural protein–protein communication processes^21^. Parallel to these efforts, much progress has been made in the ability to modify cell surfaces with synthetic agents that bear unique functionalities^28–31^. The synthetic agents can be designed to selectively target and be covalently attached to natural CSPs^32–36^. Alternatively, a synthetic anchor can be first incorporated on the cell’s surface, for example, by introducing modified saccharides to glycan chains^37–40^, unnatural amino acids^41–44^, or short peptides^45,46^ into proteins, or by creating artificial membrane-bound constructs^47–49^. The desired functionality can then be linked to these anchors using chemical^37–44,50–52^ or enzymatic reactions^45,46,53,54^. In addition, the functional group can be attached to the anchor (or to a previously introduced functional group) using non-covalent interactions, such as supramolecular host–guest interactions^55^, Watson–Crick base pairing^47,56,57^, as well as protein–protein^58,59^ or protein–DNA interactions^47,60,61^.

Inspired by recent successes in dynamically controlling protein functions and binding interactions with ODN–small-molecule conjugates^11–21^ and in cell surface engineering^28–61^, we set out to develop a dynamic artificial receptor system that combines principles from both research areas. Specifically, we hypothesized that by attaching DNA-based, stimuli-responsive protein binders on the surfaces of living bacteria, at a specific position, and by using solely self-assembly processes, a biomimetic system that imitates the dynamic features of natural CSPs can be obtained. Herein, we describe the design and operating principles of synthetic, self-assembled bacterial receptors whose structure, composition, binding interactions, and ‘expression levels’ can be reversibly controlled using external chemical signals. Importantly, we show that, similar to the responses of natural CSPs, changes that occur on the artificial receptors can alter the properties of bacteria, such as their ability to glow, adhere to surfaces, or interact with proteins and cells. Potential applications that might be achieved with such biomimetic receptor systems are also demonstrated or discussed.

## Results

### Design principles of a dynamic artificial receptor system

There is considerable interest in creating engineered bacteria and using them in various applications, such cell-based therapy and diagnosis^62^, engineering of living material^63^, and bacterial cell imaging^64^. For this reason, we selected *Escherichia coli*, the most commonly used bacteria in the biotechnology industry, to demonstrate the viability of our approach. To establish a system that imitates the dynamic features of CSPs, and that can endow *E. coli* with engineered properties, we aimed at creating artificial receptors that fulfil the following requirements: (1) The synthetic receptors would be non-covalently anchored to the surface of the bacteria. This will allow one to selectively remove them from the bacterial membrane using external molecular signals and, in doing so, control their ‘expression levels’. (2) The anchoring domain for these receptors should be stably presented on the bacterial cell surface. This will circumvent the need to re-engineer the bacteria (e.g., metabolically or genetically) prior to each modification. (3) To ensure minimal perturbation to the natural biological system and to be able to program bacterial properties in a reproducible manner, the anchoring region must be of a minimal size and be presented at a well-defined location on the bacteria membrane, respectively. Finally, (4) the synthetic receptors should be amenable to reversible modifications. This will allow us to dynamically alter their structures while attached to the bacterial membrane, akin to PTMs that occur on natural CSPs.

**Fig. 1:**
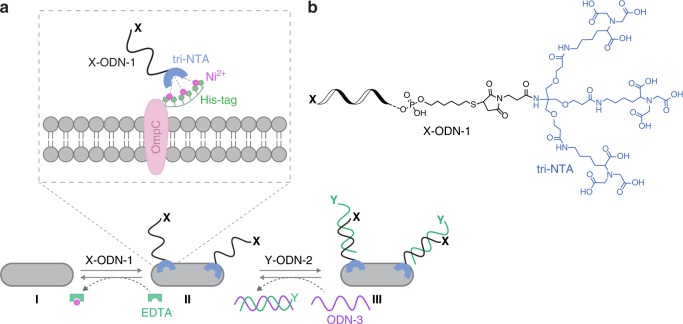
Design principles. **a** One way to decorate *E. coli* with artificial receptors, which are appended with a specific functionality (*X*), involves the binding of *X*-ODN-1 to a hexa-histidine tag (His-tag) fused to OmpC (I → II). This process can be reversed by subjecting the bacteria to EDTA (II → I). Another way to introduce an unnatural recognition motif (*Y*) to the bacterial surface is by adding to the bacteria decorated with ODN-1 a complementary strand modified with the desired functionality (*Y*-ODN-2, II → III). Y-ODN-2 can be selectively removed by adding a complementary strand, ODN-3 (III → II). **b** Structure of *X*-ODN-1.

Figure 1 shows the design and operating principles of a synthetic receptor system that fulfills these requirements. This design borrows principles from our previous studies in which we demonstrated the possibility of generating ODN–small-molecule conjugates that can non-covalently bind to several different proteins^21,22^. We hypothesized that if one of the protein targets for such synthetic protein binders would be located on the cell surface, their regulatory effect^21^ could be extended from the protein level to the cellular level. We also expected that the ability to reversibly change the structure of such DNA-based protein binders^11–22^ and precisely control the orientation, distance, and valency of their binding units^11–27^ would enable such systems to act as artificial CSPs. Namely, as artificial receptors that respond to dynamic changes in the environment and can endow the bacteria with engineered properties.

The first step in modifying bacterial surfaces with artificial receptors involves the selective binding of an ODN–small-molecule conjugate (*X*-ODN-1) to a hexa-histidine tag (His-tag) that is fused to an outer membrane protein C (OmpC) in *E. coli* (Fig. 1a, I → II). We chose OmpC as a target protein because His-tagged OmpC can be stably expressed in *E. coli*^65^ and because OmpC is one of the most abundant CSPs in this bacteria^65^, which should allow the synthetic receptors to cover a large fraction of the bacterial surface. A His-tag was selected as an anchoring domain for several reasons. First, it is one of the most common fusion tags used in cell biology, which should allow us to demonstrate the generality of the approach. Second, owing to its small size, this tag does not perturb the biological function of the protein of interest (POI). This provides the means to target His-tagged POIs in living cells without affecting the natural properties of the POI or the cell^66^. Third, because this tag can be stably expressed in *E. coli*, the bacteria could be genetically engineered only once. The fourth reason for using the His-tag is the possibility of targeting it with nitrilotriacetic acid (NTA)–nickel (II) complexes^66^, including complexes of ODN–NTA conjugates^27^. This coordination-mediated binding should allow us to remove the artificial receptors from the bacterial surface using suitable metal chelators.

The His-tag binder of *X*-ODN-1 consists of a tri-NTA group (Fig. 1b) that was previously developed in our group and has been shown to bind His-tagged proteins with low nanomolar affinity^67^. Hence, one way to decorate bacteria with artificial receptors is to modify ODN-1 with the desired recognition element (functionality *X*) and incubate the His-tagged bacteria (*E. coli* expressing His-tagged OmpC) with *X*-ODN-1 in the presence of Ni (II) ions (Fig. 1a, I → II). This process can be reversed by subjecting the modified bacteria to EDTA that can coordinate to the nickel ions and disrupt the bacteria–synthetic receptor interactions (Fig. 1a, II → I). In terms of biomimicry, this ability to change the local concentration of extracellular synthetic receptors using external molecular signals (e.g., EDTA) imitates dynamic changes that occur on CSP expression levels when the concentration of an extracellular cell signaling molecule changes.

Another, simpler way to introduce an unnatural functionality or a recognition motif (Fig. 1a, functionality *Y*) to the bacterial surface is by adding to the bacteria decorated with ODN-1 a complementary strand (ODN-2) that is modified with the desired functionality (*Y*-ODN-2, Fig. 1a, II → III). The advantage of attaching the functional group (*Y*) to ODN-2, rather than to ODN-1, is that it circumvents the synthetic complexity of combining both the tri-NTA unit and the recognition motif (*X*) on a single DNA strand (i.e., *X*-ODN-1 in step 1). Another useful property of ODN-2 is that it bears a short overhang region (“toe-hold”) known to initiate strand displacement^21^. This provides the means to selectively remove *Y*-ODN-2 from the bacteria-bound ODN-1 simply by adding a complementary strand (ODN-3). ODN-3 can hybridize with *Y*-ODN-2 (Fig. 1a, III → II), release it from the bacteria, and make ODN-1 on the surface of *E. coli* available to bind another ODN-2 that carries a different functionality.

**Fig. 2:**
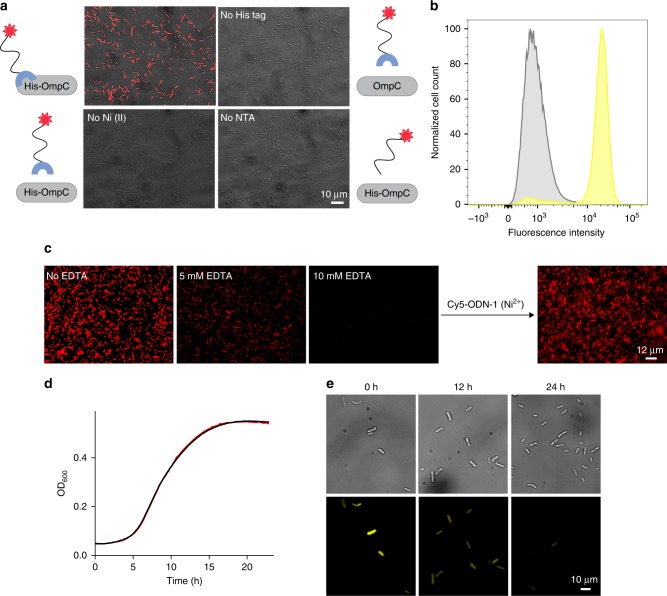
Reversible, non-covalent modification of a bacterial membrane using ODN-based synthetic receptors. **a** Merged bright-field and fluorescence images of the following: (Top left) *E. coli* expressing His-OmpC incubated with 500 nM of Cy5-ODN-1 and Ni (II). (Top right) Bacteria lacking His-tag incubated with 500 nM of Cy5-ODN-1 and Ni (II). (Bottom left) His-tagged bacteria incubated with 500 nM of Cy5-ODN-1 in the absence of Ni (II). (Bottom right) His-tagged bacteria incubated with 500 nM of Cy5-ODN (that lacks the NTA group) and Ni (II). **b** Flow cytometry analysis of His-tagged bacteria (yellow) and bacteria lacking His-tag (gray) incubated with TAMRA-ODN-1. **c** Images of *E. coli* expressing His-OmpC decorated with Cy5-ODN-1 in the presence of increasing concentrations of EDTA (0, 5, and 10 mM) (left), and following the subsequent addition of Cy5-ODN-1 in the presence of Ni (II) (right). **d** Growth curve of *E. coli* expressing His-OmpC (black) and the growth of the same bacteria decorated with TAMRA-ODN-1 (red). **e** Bright-field (top) and fluorescence images (bottom) of bacteria decorated with TAMRA-ODN-1 monitored at 0, 12, and 24 h. Source data are available in the Source Data file.

**Fig. 3:**
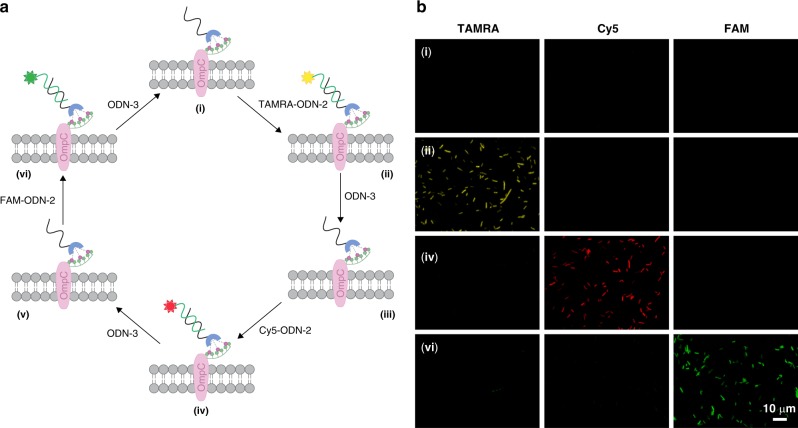
Reversible modification of synthetic receptors bound to the bacterial membrane. **a** A synthetic receptor bound to the His-tagged bacteria (ODN-1) can be sequentially modified with unnatural functionalities, here, fluorescent dyes (TAMRA, Cy5, or FAM), simply by incubating the modified bacteria with a dye-modified ODN-2 that can be selectively removed using ODN-3. **b** Monitoring states i, ii, iv, and vi in the sequential labeling process (the scheme on the left) by simultaneously observing the emissions of TAMRA, Cy5, and FAM.

**Fig. 4:**
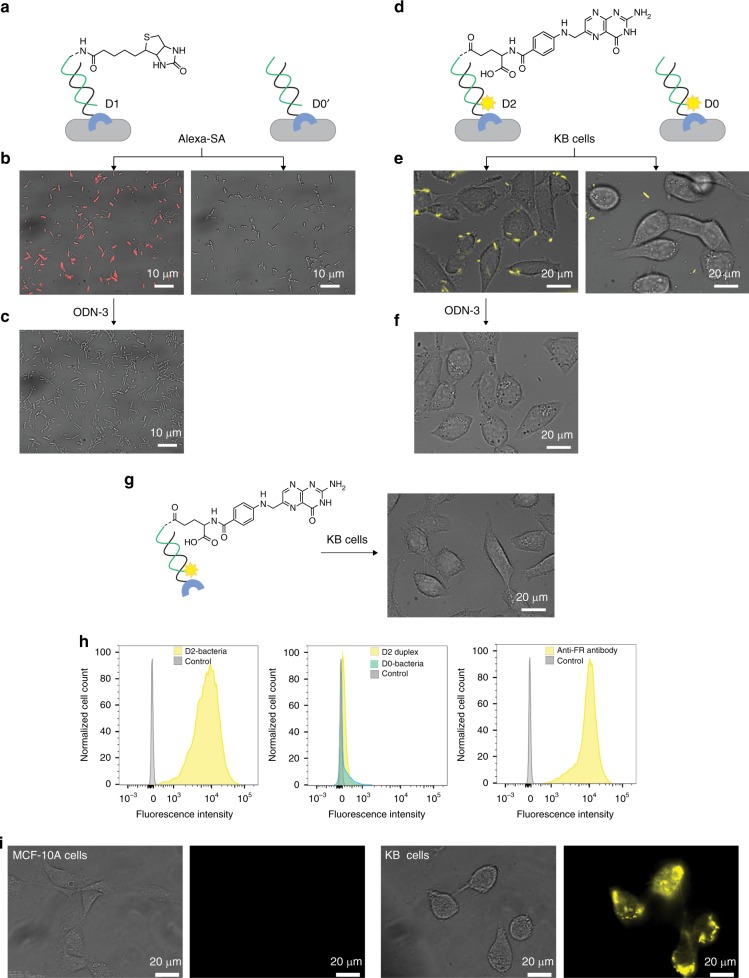
Programing the bacteria to interact with proteins and cancer cells. **a** Schematic illustration of an experiment in which modified His-tagged bacteria were treated with Alexa 647-modified streptavidin (Alexa-SA). Left: Bacteria modified with a D1 duplex generated from ODN-1 and biotin-ODN-2. Right: Bacteria modified with a D0’ duplex that lacks biotin. **b** The resulting images (merged bright-field and fluorescence). **c** Images recorded following the incubation of the bacteria bound to Alexa-SA with ODN-3. **d** Schematic illustration of an experiment in which modified His-tagged bacteria were incubated with KB-cells. Left: Bacteria decorated with a D2 duplex consisting of ODN-1 and TAMRA-labeled folate-ODN-2. Right: Bacteria decorated with a D0 duplex that lacks the folate group. **e** The resulting images. **f** Images obtained after treating the bacteria that are bound to KB cells with ODN-3. **g** Incubating KB-cells with the D2 duplex (in the absence of bacteria) did not lead to fluorescence labeling of the KB-cell. **h** Representative flow cytometry histograms of KB cells before (gray) and after (yellow/cyan) treatment with: (left) D2-modified bacteria (16 × 10^8^ cells/ml), (middle) D2 alone or D0-modified bacteria (500 nM), and (right) anti-FR antibody (0.31 μg/ml). **i** Bright field and fluorescent images of healthy MCF-10A cells (left) and cancerous KB cells (right) treated with the D2-modified bacteria.

**Fig. 5:**
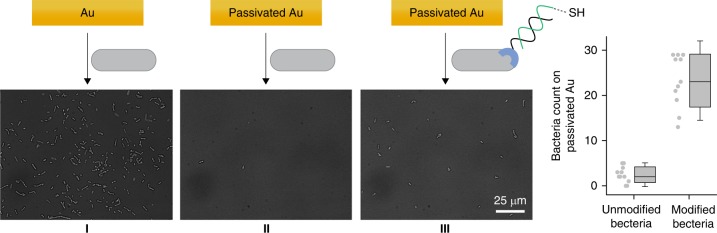
Programing the bacteria to adhere to surfaces. **a** Images of the following: (I) bare gold substrate after incubation with unmodified bacteria, (II) passivated gold substrate after incubation with unmodified bacteria, and (III) passivated gold substrate following incubation with bacteria modified with a thiol-modified duplex (ODN-1: HS-ODN-2). **b** Average number of bacteria per 0.0165 mm^2^ of passivated gold. The error bars represent the s.d. of 11 frames. Source data are available in the Source Data file.

**Fig. 6:**
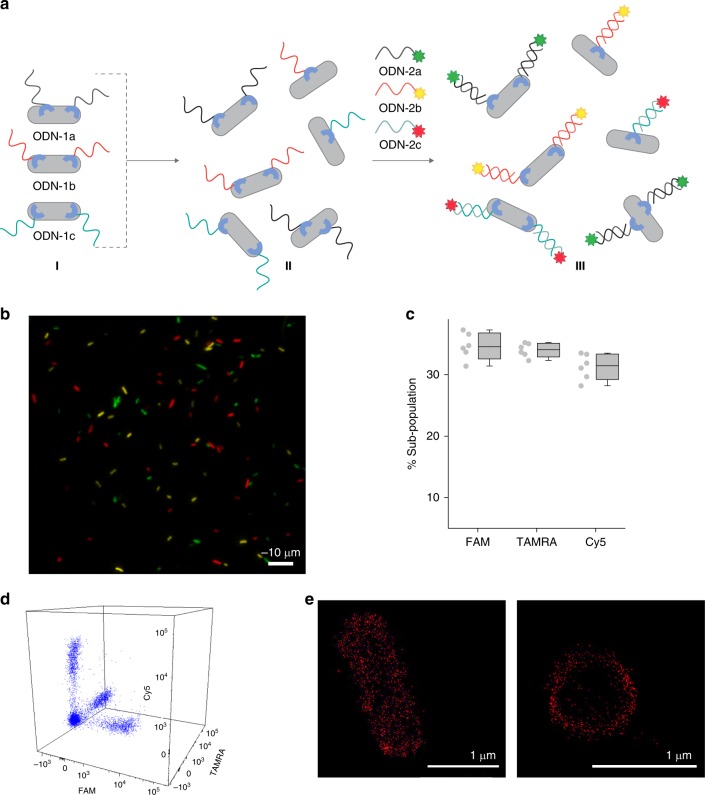
Controlling bacterial cell surface luminescence. **a** Schematic illustration of the way sub-populations of His-tagged bacteria bearing artificial receptors can be prepared (I), mixed (I → II), and selectively modified in the mixture (II → III) using ODN-2s appended with FAM, TAMRA, and Cy5. Complementary sequences are denoted in similar colors. **b** Fluorescence image of the labeled mixed population. The image corresponds to state III in the scheme. The bacteria were imaged using 488, 561, and 647 nm excitation lasers. **c** Percentage of each sub-population in the mixture. The error bars represent the s.d. of six frames. **d** Flow cytometry analysis of the mixed population. **e** STORM images of His-tagged bacteria decorated with the ODN-1:Cy5-ODN-2 duplex. Left: whole bacteria. Right: transverse cut viewed from the plane of the cell axis. Source data are available in the Source Data file.

In terms of CSP biomimicry, the bacterial surface-engineering process shown in step 2 (Fig. 1a, II → III) is conceptually different from the one discussed in step 1 (I → II). Step 1, in which the local concentration of bacteria-bound synthetic receptors can be reversibly controlled, imitates changes in the CSP expression levels. On the other hand, introducing a different structural motif to a synthetic receptor that is already bound to the bacteria (ODN-1) (step 2) resembles PTMs that occur on natural CSPs. With this perception in mind, we synthesized sets of modified ODN-1s and ODN-2s and used them to demonstrate the underlying design principles (Figs. 2 and 3). In addition, these ODNs were used to demonstrate the way these artificial receptors can endow bacteria with unnatural properties that might be useful for future applications (Figs. 4–6).

### Controlling artificial receptors ‘expression levels’

To demonstrate the first method for reversibly modifying bacterial membranes with synthetic agents (Fig. 1a, I → II), OmpC modified with three hexa-histidine repeats (His-OmpC) was expressed in *E. coli* and the engineered bacteria were incubated with ODN-1 appended with Cy5 (Fig. 2a, Cy5-ODN-1) in the presence of nickel ions. Fluorescence imaging revealed that the His-tagged bacteria were fluorescently labeled (Fig. 2a, top left), as expected from our design. To confirm that the labeling did not result from non-specific interaction between Cy5-ODN-1 and the bacterial surface, Cy5-ODN-1 was also incubated with bacteria that lack the His-tag (Fig. 2a, top right), as well as with the His-tagged bacteria in the absence of nickel ions (Fig. 2a, bottom left). In addition, the His-tagged bacteria were incubated with a Cy5-labeled ODN that lacks a tri-NTA group (Fig. 2a, bottom right). The fact that the fluorescent labeling occurred only when Cy5-ODN-1, His-tagged *E. coli*, and nickel ions were combined (Fig. 2a, top left) confirms the selectivity of ODN-1 toward the His-tag on the bacterial membrane. The selectivity and degree of labeling were further analyzed by flow cytometry (Fig. 2b), which revealed 1% (gray) and 90.9% (yellow) labeling of the bacteria expressing OmpC and His-OmpC, respectively. As discussed before, a unique feature of this biomimetic system is the ability to control the expression levels of the artificial CSPs with an external chemical signal (Fig. 1a, II → I). As shown in Fig. 2c, exposing bacteria labeled with Cy5-ODN-1 to increasing concentrations of EDTA resulted in a gradual decrease in emission, which results from a decrease in synthetic receptor coverage. Moreover, with 10 mM EDTA, Cy5-ODN-1 was completely removed, which allowed us to decorate the bacteria with Cy5-ODN-1 one more time (Fig. 2c, right).

To enable practical use of this biomimetic CSP system, it is critical to ensure that the bacteria remain viable following modification. To this end, we followed the growth of His-tagged bacteria decorated with TAMRA-ODN-1 by recording optical density (OD) over time. The growth curve (Fig. 2d, red line) was compared to the one obtained for the bare His-tagged bacteria (Fig. 2d, black line). The fact that the growth rate was not affected by the binding of TAMRA-ODN-1 to His-tagged OmpC indicates that the biomimetic CSP system neither induces toxicity nor disrupts cell division. The ability of the labeled bacteria to grow and divide was further demonstrated using fluorescence microscopy (Fig. 2e). Because the bacterial membrane is divided between the two daughter cells during division, we expected that the TAMRA-ODN-1 strand would stay bound to the membrane during division; however, its number per cell will decrease with every division. The increase in the number of labeled cells over time (Fig. 2e, top) and the simultaneous decrease in fluorescence from each cell (Fig. 2e, bottom) confirmed this hypothesis.

### ‘PTMs’ of the synthetic receptors

The ability to dynamically alter the functionality attached to the bacteria-bound artificial receptors (Fig. 1, II → III), in a way that resembles PTMs, was demonstrated by subjecting His-tagged *E. coli* modified with ODN-1 to a fluorescently labeled ODN-2 (TAMRA-ODN-2, Cy5-ODN-2, or FAM-ODN-2) in the presence of Ni (II), and then, to ODN-3, which can induce strand displacement (Fig. 3a). Imaging the bacteria following sequential incubation with TAMRA-ODN-2, ODN-3, Cy5-ODN-2, ODN-3, and FAM-ODN-2 (Fig. 3b) revealed that in the presence of any of the dye-labeled ODN-2s the bacterial membrane becomes fluorescent and that this emission disappears following the addition of ODN-3, as expected from our design (Fig. 3a). Although this study focuses on reversible modification of synthetic receptors, the use of ODNs to scaffold these receptors opens the way to irreversibly modify them by using a range of DNA-modifying enzymes (e.g., ligases and nucleases). To demonstrate this possibility, we imaged the Cy5-ODN-1-modified bacteria following incubation with benzonase (Supplementary Fig. 1). The disappearance of the emission, indicates cleavage of the membrane-bound synthetic receptors by the nuclease and thus, the ability to achieve additional types of artificial PTMs by means of enzymatic engineering.

### Artificial receptors as a means to alter bacterial properties

In nature, various properties of bacteria are mediated by structural changes and binding interactions that occur on CSPs^1–10^. By changing the functional groups (*Y*) on *Y*-ODN-2 (Figs. 4–6), we demonstrate the possibility of using the artificial receptor system to endow the bacteria with unnatural and potentially useful properties.

### Unnatural interactions with proteins and cells

The binding of cells to extracellular proteins or to other cells is mediated by highly specific and reversible interactions between extracellular receptors and their protein-binding partners. To mimic these features with our artificial receptor system, we modified His-tagged *E. coli* with synthetic receptors (ODN-1:*Y*-ODN-2 duplexes) appended with a specific protein binder (*Y*) and investigated whether such receptors can endow the bacteria with the ability to engage in unnatural binding interactions. Initially, the His-tagged bacteria were decorated with a duplex containing a biotin-modified ODN-2 (Fig. 4a, left, D1) and the modified cells were incubated with an Alexa 647-modified streptavidin (Alexa-SA). To verify specificity, the same experiment was performed with a similar duplex that lacks biotin (Fig. 4a, right, D0′). Inspecting the fluorescence images obtained following treatment with the fluorescent streptavidin (Fig. 4b) showed that the bacteria became fluorescent only when biotin-ODN-2 was incorporated in the synthetic receptor (Fig. 4b, left), indicating specific binding of the protein to the bacterial membrane. The disappearance of the emission when ODN-3 was added (Fig. 4c) indicates the ability to reverse this process and consequently, that it is possible to regulate unnatural cell–protein interactions using synthetic molecular signals (e.g., biotin-ODN-2 and ODN-3). In the next step, we investigated whether synthetic receptor–protein interactions could also be used to mediate unnatural cell–cell interactions. In particular, we checked whether the biomimetic system could induce interactions between bacteria and mammalian cells, in a way that imitates host cell recognition during infection^3^. To this end, His-tagged bacteria were decorated with a DNA duplex containing ODN-1 and a folate-modified ODN-2 (Fig. 4d, left, D2) and were incubated with human epidermoid carcinoma KB cells overexpressing an extracellular folate receptor (FR). As a control, KB cells were also incubated with bacteria decorated with the D0 duplex that lacks the folate group (Fig. 4d, right). Imaging the KB cells following incubation and washing (Fig. 4e) revealed that only the folate-modified ODN-2 induces an unnatural interaction between the His-tagged bacteria and the mammalian cells, resulting in cancer cells being clearly labeled with the fluorescent bacteria (Fig. 4e, left). The imaging also showed that ODN-3 can remove the synthetic FR binder from the bacteria after binding to the KB cells (Fig. 4f), indicating that unnatural cell–cell interactions can be both induced and disrupted using a biomimetic receptor system that responds to external molecular signals (i.e., folate-ODN-2 and ODN-3).

Interestingly, unlike the fluorescence labeling of KB cells with the folate-modified bacteria (Fig. 4e, left), incubating the KB cells with the folate-modified DNA duplex alone (not bound to the His-tagged bacteria) did not lead to fluorescent labeling of the cancer cells (Fig. 4g). This observation indicates that the bacterial scaffold itself plays a critical role in the labeling process (Fig. 4e, left). One contribution of the bacterial scaffold to the effective cell labeling (Fig. 4e, left) is increased avidity, which results from multivalent interactions between the FRs on the KB cell and multiple folate-modified DNA duplexes on the surface of *E. coli*. The second contribution is that each bacterial cell is decorated with multiple fluorophores and hence, a much stronger fluorescent signal can be locally detected when a fluorescently labeled bacterium binds to FRs on the cancer cell. Another possible reason for the improved labeling is that the binding of the labeled bacterial scaffold to human cells induces additional inter-cellular interactions (e.g., interactions between bacterial adhesins to CSPs of KB cells), which are not mediated by the FRs.

Realizing that the bacterial scaffold can significantly enhance both the affinity and brightness of fluorescent molecular probes has stimulated us to determine whether the modified bacteria could serve as biosensors for the selective identification of diseased cells. Initially, flow cytometry was used to determine the degree of labeling of the entire cancer cell population with the D2-modified bacteria (Fig. 4h, left), as well as with the D2 duplex alone or the D0-modified bacteria (Fig. 4h, middle). In addition, we compared the degree of labeling with the ‘living probe’ (Fig. 4h, left) to the one obtained with a conventional antibody-based probe (i.e., a fluorescent anti-FR-Ab) (Fig. 4h, right and Supplementary Fig. 2). FACS analysis confirmed that the D0-bacteria do not label the KB cells (Fig. 4h, middle). More importantly, it showed that attachment of D2 to the bacteria increased the percentage of labeling from 0% (Fig. 4h, middle) to 100% (Fig. 4h, left) and that obtaining a similar labeling efficiency with immunofluorescence (Fig. 4h, right) requires using ~1000-fold more probe concentration. The diagnostic capabilities of the living probes were further demonstrated by subjecting healthy cells (Fig. 4i, left) and cancer cells (Fig. 4i, right) to the folate-modified bacteria, which resulted in selective labeling of the latter.

Taken together, these experiments indicate the potential to develop a class of fluorescent probes for cancer diagnosis that may complement conventional methods, such as immunofluorescence. One benefit of using such systems over immunofluorescence is that they circumvent the need to use antibodies (Abs). Whereas Abs are costly to produce and have limited shelf-life, the living probes are generated from synthetic receptors, which are stable and easier to make, and from bacterial scaffolds that can self-multiply and be stored for decades. The advantage of using our system over other types of chemically engineered cells is the ability to generate the modified bacteria by simple self-assembly processes. As a result, the living and synthetic components can be separately prepared and stored and can be combined only prior to the diagnostic procedure without having to use complex experimental protocols and trained personnel.

These last experiments (Fig. 4d–i) also demonstrate, at the proof-of-principle level, the relevance of this study to cell-based therapy. Currently, there is a wide interest in using genetically modified mammalian and bacterial cells as therapeutics^62^. Therefore, the ability to program bacterial cells to target cancer cells with increased avidity and selectively indicates the possibility of using synthetic cell-surface receptors to guide therapeutic cells to their targets. Disrupting the unnatural bacteria–cancer cell interactions with ODN-3 (Fig. 4f) demonstrates the potential to develop antidotes for this class of therapeutics.

### Unnatural adhesion to solid support

Unlike the specific interactions with proteins or cells, adhesion of bacteria to abiotic surfaces is mediated by cell surface adhesins that form non-specific interactions with solid supports^2,3^. For example, incubating gold surfaces with unmodified bacteria leads to dense coverage of the surface with bacterial cells (Fig. 5a–I) owing to non-selective adhesin–gold interactions. In the following experiments (Fig. 5a-II and 5a-III) we show that, in addition to serving as synthetic receptors that form specific interactions with proteins or cells (Fig. 4), the artificial CSPs can also function as unnatural adhesins that enable the bacteria to selectively interact with solid substrates.

To prevent non-specific interactions between the bacteria and the surface, the gold substrate was modified with a passivation layer of (11-mercaptoundecyl)tetra(ethylene glycol), which has been shown to prevent non-specific bacterial adhesion^68^. As shown in Fig. 5a-II, incubation of the passivated gold slides with unmodified bacteria led to negligible bacterial attachment. The ability to program bacteria to selectively attach to the same substrate was demonstrated by subjecting the passivated surface to *E. coli* decorated with a DNA duplex assembled from ODN-1 and HS-ODN-2, namely, an ODN-2 that is appended with a thiol group (Fig. 5a-III). The latter is known to have high affinity toward gold. Imaging the surface (Fig. 5a-III) revealed ~10-fold increase in the attachment of thiol-modified bacteria to the gold substrate compared with the unmodified bacteria (Fig. 5b, III vs. II), indicating that the ODN-1:HS-ODN-2 duplex acts as an unnatural adhesin that mediates the specific binding of *E. coli* to solid support. Although the focus of this experiment is extending the biomimetic nature of our approach by demonstrating an additional means to program bacterial behavior, obtaining unnatural bacterial adhesion also indicates the relevance of this study to technologies focusing on attaching living cells to solid supports with well-defined architectures^50–52^. The unnatural adhesins used in this study do not require the cells to be chemically modified at random positions on the cell surface, nor that they be metabolically engineered prior to each modification. Because these adhesins can reversibly bind to a very short and well-defined anchor (i.e., His-tag), which can be stably expressed on a specific membrane protein (i.e., OmpC), future generations of such adhesins may contribute to studying the biophysical properties of bacteria on solid support, as well as to generating engineered living materials (ELMs) or devices with controlled properties.

### Unnatural Luminescence

Reversible switching of luminescence in response to the binding of CSPs to extracellular molecular signals (i.e., AIs) is a fundamental property of several bacterial strains^4^. Therefore, in addition to validating the operating principles of our system, the previous experiments, in which bacterial membrane fluorescence could be reversibly switched (Figs. 3 and 4) using synthetic molecular signals (dye-ODN-1 and EDTA or dye-ODN-2 and ODN-3), demonstrated the potential to control bacterial cell luminescence using a synthetic receptor system. A key principle underlying natural bacterial luminescence processes is the selective interaction between protein receptors and extracellular signals, which enables the latter to trigger the emission of specific bacterial strains in complex biological mixtures^4^. To demonstrate that such high selectivity can also be obtained with our biomimetic receptor system, three distinct populations of modified bacteria were prepared by incubating identical samples of His-tagged *E. coli* with three different types of ODN-1 (ODN-1a, ODN-1b, and ODN-1c), namely, with ODNs bearing the same tri-NTA group but that differ in their sequences (Fig. 6a, state I). Then, the three samples were combined to afford a mixed population of synthetic receptor-modified bacteria (Fig. 6a, state II) and the mixture was subjected to a mixture of external inputs (ODN-2a, ODN-2b, and ODN-2c), namely, FAM-, TAMRA-, and Cy5-labled ODN-2 (Fig. 6a, II → II), each of which complements only one of the bacteria-bound ODN-1s. We reasoned that if each sub-population in the mixture would only bind its designated inputs, then the bacterial cell mixture would consist of only three types of glowing bacteria, where each type fluoresces with a unique emission color (Fig. 6a, state III). Imaging the bacteria (Fig. 6b) and analyzing the composition of the labeled bacteria in these images (Fig. 6c) and by using FACS (Fig. 6d), showed the formation of three distinct populations, where each population is labeled with only one dye. Calculating the percentage of each population out of the total number of bacteria revealed a 1:1:1 ratio between the three sub-populations. This indicates that there is no strand swap between the three populations and thus, that the sub-population modification occurs with very high selectivity. Note that, unlike with natural bacterial luminescence, the emission observed in this experiment does not result from the activation of a cell-signaling pathway. Hence, in terms of biomimicry, the current system demonstrates how selective bacteria recognition leads to a specific fluorescence emission, but not to an artificial signal transduction step.

Beyond biomimicry, this experiment demonstrates a means to selectively label His-tagged proteins with different colors. Hence, one practical application that can be achieved with this approach is using the synthetic receptors to image specific proteins in living cells. The advantage of using this method over using other fluorescent probes that can bind and label short fusion peptides in living cells^28^ is the simplicity by which the fluorescent dye can be changed. Specifically, when DNA-based fluorescent probes are used for live cell imaging^69,70^, there is no need to synthesize a new probe for each application. Instead, various different fluorescent dyes can be used for imaging, simply by preparing a wide range of fluorescently labeled ODNs from commercially available phosphoramidites and by using an automated DNA synthesizer. To demonstrate the practicality of this approach for live cell-imaging applications, we used it to visualize *E. coli’s* membrane with super resolution (SR) (Fig. 6e). Owing to the small size of bacteria (1–10 µm), conventional fluorescence microscopes are generally unsuitable for imaging and therefore, methods for visualizing bacterial membranes with SR microscopy are highly desired^64^. *E. coli*’*s* membrane was imaged in high resolution (Fig. 6e) simply by combining ODN-1 with a commercially available ODN-2 (Cy5-ODN-2) bearing a Cy5 dye, which is compatible with stochastic optical reconstruction microscopy (STORM). STORM images of individual bacteria (Fig. 6e, left) revealed that the DNA duplex-based label clearly outlines the bacterial cell’s borders. The transverse cut of the bacteria (Fig. 6e, right) confirms that only the outer membrane of the bacteria is labeled, namely, that the synthetic receptors are exposed on the bacterial surface and are not internalized.

## Discussion

The advantages of using ODN–small-molecule conjugates as synthetic protein binders include the ability to precisely control the orientation, distances, and valency of their binding units^11–27^, as well as the ability to dynamically change their structure, which provides a means to regulate protein functions in real time^11–21^. This work shows that when synthetic protein binders of this class are attached to cell surfaces, their regulatory effect can be extended from the protein level to the cellular level. Specifically, on the cell membrane such systems can act as artificial cell surface receptors that can be reversibly modified; hence, they can provide the cells with programmable properties. In this model system, metal coordination and DNA-hybridization were used to direct the formation of artificial receptors on a short peptide tag fused to an outer membrane protein on the surface of *E. coli*. Owing to the high selectivity and reversibility of the self-assembly processes, a biomimetic cell surface receptor system with unique features was obtained. For example, the ability to reversibly control the type of membrane-bound receptors and their local concentration levels using external molecular signals demonstrates the possibility of imitating dynamic processes that occur with CSPs, such as changes in their expression level or PTMs. We have also shown that these changes can provide the bacteria with new properties, such as an ability to glow with different colors, adhere to surfaces, and interact with proteins or cells, properties that may eventually be used in developing cell-imaging methods, living materials, and devices, as well as live cell-based therapeutics and diagnostic systems, respectively. A notable distinction between the biomimetic receptor system and various natural receptors is that the latter exert their biological effect by activating a signal transduction step. We therefore hope that this study will encourage the development of additional types of biomimetic receptors, capable of inducing unnatural cell-signaling events. We also expect that in light of the various potential applications demonstrated in this work, additional biomimetic cell surface receptors, with which living cells could be programed to perform diverse sets of functions, will be developed.

## Methods

### Bacterial strains and growth conditions

*E. coli* K-12 strain KRX (Promega) was used for protein expression. Transformed bacteria with different OmpC constructs (OmpC or His-OmpC) were cultured to saturation in LB medium supplemented with 100 μg/ml of ampicillin at 30 °C. Next, pre-cultured cells were diluted 1:100 in fresh LB medium supplemented with ampicillin, and incubated until the OD_600_ reached ~0.6. Protein expression was then induced by the addition of 0.1% Rhamnose and 20 μM isopropyl-b-d-1-thiogalactopyranoside (IPTG) and cultures were allowed to grow at 30 °C for 18 h.

### Decorating bacteria with the oligonucleotides

The bacterial cells (OmpC or His-OmpC) were collected by centrifugation at 6000 × *g* for 4 min. Pellets were washed twice with PBS × 1 buffer and resuspended in the same buffer to an OD_600_ of 0.3. To a 100 μl sample of the bacteria suspension, a preincubated sample of DNA (500 nM) and NiCl_2_ (2.5 μM) was added, and the cells were incubated at room temperature for 1 h. Then the bacterial sample was washed twice with PBS, resuspended in 100 µl PBS, and placed on a glass-bottom dish (P35G-1.5-14-C; MatTek) precoated with poly-l-lysine (Sigma Aldrich) and left to adhere for 1 h. Finally, the wells were washed vigorously with PBS three times and imaged using an Olympus IX51 fluorescent microscope. The samples were imaged using ×60 or ×100 objective lenses.

### Treating the modified bacteria with EDTA

Bacterial samples decorated with Cy5-ODN-1 were incubated with various concentrations of EDTA (0, 5, 10 mM) for 1 h. Cells were then collected (6000 × *g*, 4 min) and washed twice with 200 μl PBS buffer. Finally, cells were resuspended in 100 μl PBS buffer and added to poly-l-lysine-coated slides for imaging.

### Flow cytometry analysis of bacteria labeling

His-tagged bacteria and bacteria lacking His-tag decorated with TAMRA-ODN-1 were analyzed and sorted on a BD FACSAria Fusion instrument (BD Immunocytometry Systems) equipped with a 405, 488, 561, and 640 nm lasers, using a 100 µm nozzle, controlled by BD FACS Diva software v8.0.1 (BD Biosciences), at the Weizmann Institute of Science Flow Cytometry Core Facility. Further analysis was performed using FlowJo software v10.2 (Tree Star).

### Flow cytometry analysis of cancer cell labeling

KB cells were maintained in folate-depleted RPMI supplemented with 10% fetal bovine serum (FBS), 1% l-glutamine, and 1% penicillin/streptomycin. KB cells were counted using an automated cell cytometer and samples of 1 × 10^6^ cells per Eppendorf tube were prepared. To determine the minimal amount of living probe needed to obtain substantial KB cell labeling, the cells (1 × 10^6^) were incubated with increasing concentrations of His-tagged bacteria (2.5 × 10^7^–16 × 10^8^ cells/ml) decorated with the ODN-1:folate-ODN-2 duplex (D2-duplex) at 37 °C for 15 min. Cells were centrifuged (380 × *g* for 3 min) and washed twice with PBS. Finally, cells were re-suspended in FACS buffer (1% BSA in PBS), and analyzed by flow cytometry. For the control experiment, KB cells (1 × 10^6^) were incubated with bacteria (16 × 10^8^ cells/ml) decorated with a D0 duplex consisting of ODN-1 and ODN-iii (Supplementary Information) or with the D2-duplex (500 nM). To determine the amount of Abs required to obtain a comparable signal intensity, cells were incubated with increasing concentrations (0.0195–0.31 μg/ml) of PE anti-FoR1 antibody (Biolegend Cat. No. BLG-908303, dilutions of 1:80, 1:160, 1:320, 1:640 and 1:1280, respectively) or PE mouse IgG2a, κ isotype control (1.25 μg/ml, Biolegend Cat. No. BLG-400213, dilution of 1:160) at 4 °C for 30 min. Then the cells were washed twice, re-suspended in FACS buffer, and analyzed by FACS. The bacteria:Ab ratio (1:1000) was determined by calculating the number of bacterial cells and Abs according to the OD at 600 nm (OD_600_) and a molecular weight of 150 kDa, respectively.

### Bacterial cell growth

His-OmpC bacteria decorated with TAMRA-ODN-1 were incubated for 30 min in M9 minimal medium containing 2% glucose. The sample was spun down at 6000 × *g* for 2 min and the supernatant was discarded. After the pellet was washed with M9 minimal medium, the cells were diluted to OD_600_ = 0.05 in M9 medium in a 96-well plate. Growth kinetics was monitored by recording OD_600_ with shaking at 30 °C for 24 h. Bacteria expressing His-OmpC were used as a control. The ability of the modified His-tagged bacteria to grow and divide was also demonstrated using fluorescence microscopy. For these experiments, the bacteria were prepared using a similar procedure. After the sample was diluted to OD_600_ = 0.3, it was allowed to grow at 30 °C. Next, 100 µl samples were withdrawn at different time intervals, and plated on poly-l-lysine-coated glass bottom dishes and imaged by fluorescent microscopy.

### Reversible modification of synthetic receptors

Bacterial cells decorated with ODN-1 were washed with PBS, and the following ODNs were added sequentially: TAMRA-ODN-2, ODN-3, Cy5-ODN-2_,_ ODN-3, FAM-ODN-2_,_ and ODN-3. After each incubation step, cells were washed twice with PBS and a sample was taken for imaging before adding the subsequent strand. Fluorescently labeled ODN-2 strands were added at a concentration of 500 nM and incubated for 30 min, while the ODN-3 strand was added at a concentration of 2 µM and incubated for 2 h.

### Irreversible modification of synthetic receptors

Bacterial cells decorated with Cy5-ODN-1 were incubated with benzonase nuclease (Millipore, 12.5 U) for 0.5 and 1.5 h. The bacterial samples were then washed twice with PBS, re-suspended in 100 µL PBS, and placed on poly-l-lysine glass-bottom dishes. Finally, the wells were washed vigorously with PBS three times and the samples were imaged using ×60 objective lenses.

### Preparation of mixed bacterial populations

Three samples of His-OmpC bacteria (100 µl each) were separately labeled with ODN-1a, ODN-1b, or ODN-1c. Each sample was washed twice with PBS. Then, an equal ratio (30 µl each) of the three samples was combined and ODN-2a, ODN-2b, and ODN-2c (500 nM) were added to the mixture and incubated for 10 min. The bacterial cells were centrifuged at 6000 × *g* for 2 min, washed twice with PBS, and imaged by fluorescent microscopy using 488, 561, and 647 nm excitation lasers. For flow cytometry analysis, the samples were not washed after adding ODN-2 strands.

### Fluorescent imaging of bacteria–streptavidin interaction

His-tagged bacterial cells were decorated with a duplex consisting of ODN-1 and biotin-ODN-2 duplex. For binding with streptavidin, cells were incubated with Alexa-647 streptavidin conjugate (500 nM) in PBS buffer for 1 h, and after having been washed twice with PBS, they were imaged by fluorescent microscopy. The fluorescent signal was abolished when bacterial cells were treated with ODN-3 (3 μM) for 1 h. The control experiment was performed similarly using bacteria decorated with a duplex containing ODN-1 and the complementary strand.

### Fluorescent imaging of bacteria cell interaction

KB cells were maintained in folate-depleted RPMI supplemented with 10% FBS, 1% l-glutamine, and 1% penicillin/streptomycin. MCF-10A cells were maintained in phenol-free Dulbecco’s Modified Eagle Medium (DMEM/F-12) supplemented with 5% horse serum, 1% l-glutamine, 1% penicillin/streptomycin, 10 ng/ml EGF, 10 mg/ml insulin, 0.5 mg/ml hydrocortisone, and 0.1 mg/ml cholera toxin. Cells (12,500 cells/well) were seeded onto glass bottom culture dishes (Mattek) and allowed to adhere overnight. Cells were then washed twice with PBS and incubated with 100 µl His-tagged bacteria decorated with ODN-1:folate-ODN-2 duplex for 30 min. The medium was removed and cells were rinsed three times with PBS. Then cells were imaged using a fluorescence microscope and a ×60 objective lens. A control experiment was performed similarly using bacteria decorated with a duplex lacking the folate moiety (ODN-1 and ODN-iii). To show the reversibility of the interaction, the bacteria-bound KB cells were incubated with ODN-3 (5 μM) for 15 min. After having been washed twice with PBS buffer, cells were imaged again.

### Adhesion to the solid support

The gold substrates were prepared by electron-beam evaporation of an adhesion layer of chromium (3 nm), followed by a 20 nm layer of gold (99.99% purity) onto high-precision cover glasses (170 ± 5 μm, Marienfeld-Superior, Germany). A solution of (11-mercaptoundecyl)tetra(ethylene glycol) (2 mM in ethanol) was added to the gold-coated substrates and incubated for 2 h. After the solution was removed, the slides were washed four times with ethanol. Bacterial samples decorated with a duplex consisting of ODN-1 and HS-ODN-2 were washed twice with PBS, resuspended in 100 µl phosphate buffer (pH = 3.8), and then incubated on gold surfaces for 15 min. The solution containing bacteria was removed, and the slides were rinsed three times with PBS, and twice with water. Finally, they were imaged using an Olympus IX51 microscope.

### Super-resolution microscopy

Super-resolution images were collected on a Vutara SR200 STORM (Bruker) microscope based on single-molecule localization biplane technology. His-tagged bacteria were decorated with ODN-1:Cy5-ODN-2 duplex were imaged using a 647 nm excitation laser and a 405 nm activation laser in an imaging buffer composed of 5 mM cysteamine, oxygen scavengers (7 μM glucose oxidase and 56 nM catalase) in 50 mM Tris, 10 mM NaCl, and 10% glucose at pH 8.0. Images were recorded using a ×60 NA 1.2 water immersion objective (Olympus) and an Evolve 512 EMCCD camera (Photometrics) with gain set at 50, the frame rate at 50 Hz, and a maximal power of 647, and 405 nm lasers were set at 6 and 0.05 kW/cm^2^, respectively. The total number of frames acquired was 8000. Data were analyzed by Vutara SRX software. SR microscopy was performed at the Irving and Cherna Moskowitz Center for Nano and Bio-Nano Imaging at the Weizmann Institute of Science.

### Oligonucleotide synthesis and OmpC expression

Further information on synthesis and characterization of the oligonucleotides and OmpC expression is available in the Supplementary Information section.

### Reporting summary

Further information on research design is available in the Nature Research Reporting Summary linked to this article.

## Supplementary information

Supplementary Information

Reporting Summary

## Data Availability

The data supporting this study are available in the Supplementary Information or from the corresponding author upon request. Source data for Figs. 2, 5, and 6 as well as Supplementary Figure 2 are available in the Source Data file.

## Source data

Source Data
