# Supplementary material for: Decorating bacteria with self-assembled synthetic receptors
Source: Nat Commun. 2020 Mar 10;11:1299. doi: 10.1038/s41467-020-14336-7 (PMC7064574; doi:10.1038/s41467-020-14336-7)
Supplement: Supplementary file 1 — Supplementary Information [file 41467_2020_14336_MOESM1_ESM.pdf]

Supporting Information

Decorating Bacteria with Self-Assembled Synthetic Receptors

Lahav-Mankovski N et al.

1. Materials and methods

All reagents and solvents were obtained from commercial suppliers. Oligodeoxynucleotides (ODNs) were obtained from W. M. Keck Foundation Biotechnology at Yale University. Alexa-647 Streptavidin conjugate was a kind gift from Prof. Roy Bar-Ziv. Aluminum-backed silica plates (Merck silica gel 60 F254) were used for thin layer chromatography (TLC) to monitor solution-phase reactions. The ^{1}H-NMR spectra were recorded using a 300 MHz Bruker Avance NMR spectrometer. Chemical shifts are reported in ppm on a δ scale down field from TMS as the internal standard. The following abbreviations were used to describe the peaks: s-singlet, d-doublet, t-triplet, q-quartet, quin-quintet, and m-multiplet. Electronspray mass spectrometry was performed with a Micromass Platform LCZ-4000 instrument at the Weizmann Institute of Science mass spectrometry facility. Matrix-assisted laser desorption ionization time-of-flight (MALDI-TOF) mass spectrometry was performed on an AB SCIEX 5800 system, equipped with an Nd: YAG (355 nm) laser with a 1 KHz pulse (Applied Biosystems), at the Weizmann Institute of Science mass spectrometry facility. The purification of oligonucleotides was carried out on a Waters 2695 separation module HPLC system with a 2994 photodiode array detector using either a Waters XBridgeTM OST C18 column (2.5 µM, 4.6 mm × 50 mm) or an XBridgeTM OST C18 column (2.5µM, 10 mm × 50 mm). The purified ODNs were analyzed by analytical HPLC and were found to have a purity of >97%. Oligonucleotide samples were desalted using illustra MicroSpin G-25 Columns (GE Healthcare) according to the supplier's instructions. Concentrations of the oligonucleotides were quantified based on their respective electronic absorption at 260 nm and the molar extinction coefficient of the oligonucleotide at this wavelength. KB and MCF-10A cell lines were obtained from Prof. Ronit Satchi-Fainaro’s group (Tel Aviv University, Israel) and Prof. Yosef Yarden’s group (Weizmann Institute of Science, Israel), respectively. These cell lines were screened negative for mycoplasma using a PCR-based assay (EZ-PCR mycoplasma detection kit, Biological Industries). Cell images were acquired using an Olympus IX51 fluorescent microscope equipped with a U-MNIBA3 fluorescence filter cube (excitation and emission filters of 470-495 nm, and 510-550 nm, respectively), a U-MNG2 fluorescence filter cube narrow-band (excitation and emission filters of 530-550 nm, and 590 nm, respectively) and a U-MF2 fluorescence filter cube (excitation and emission filters of 620-660 nm, and 700-775 nm, respectively).

2. Synthetic procedures

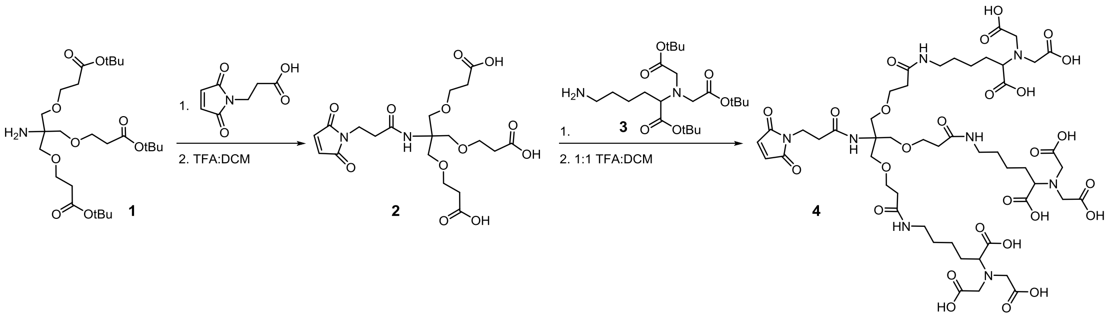

Compounds **1** and **3** were synthesized according to previously reported procedures.[1, 2]

Compound 2: Compound **1** (600 mg, 1.18 mmol) was dissolved in dry DCM (30 ml) under argon and cooled to 0°C. Then, EDC (339 mg, 1.7 mmol) and DIPEA (413.7 µl, 2.32 mmol) were added and the reaction mixture was stirred for 30 min at room temperature. 3-Maleimidopropionic acid (240.1 mg, 1.4 mmol) was added, and the solution was stirred overnight. Then 40 ml DCM was added and the solution was washed with water (10 ml), and brine (10 ml). The organic layer was dried with Na_2SO_4, filtered, and concentrated under high vacuum. Finally, the crude product was purified by column chromatography (DCM/MeOH, 97:3) to yield a yellow oil (501.6 mg, 64%). ^{1}H NMR ($CDCl_3$, 300 MHz): δ 1.44 (s, 27H); 2.44 (t, J = 6 Hz, 6H); 2.51 (t, J = 6 Hz, 2H); 3.63 (t, J = 6 Hz, 6H); 3.67 (s, 6 H); 3.80 (t, J = 6 Hz, 6H); 6.69 (s, 2H). ESI-MS (m/z): calcd. for (M+H): 657.35, found 657.44; calcd. for (M+Na): 679.35, found 679.31. The tert-butyl groups were then deprotected using a 1:1 (v/v) mixture of TFA: DCM for 2.5 h. After the solvents were removed, the excess of TFA was co-evaporated 4 times with DCM and then the product was dried under high vacuum. ^{1}H NMR (D_2O, 300 MHz): δ 2.47 (t, J = 6 Hz, 2H); 2.59 (t, J = 6 Hz, 6H); 3.61 (s, 6H); 3.67-3.75 (m, 8H); 6.83 (s, 2H). ^{13}C NMR ($(CD_3)_2SO$, 125 MHz) δ 34.0, 34.7, 48.7, 59.8, 66.8, 68.2, 134.6, 169.8, 170.9, and 172.8. HRMS-ESI$^+$ (m/z) calcd. for $[M+H]^+$, 489.1715 found, 489.1719.

Compound 4: A solution of compound **2** (160 mg, 304.8 µmol) in dry DCM (10 ml) was cooled to 0 °C in an ice bath and DIPEA (212 µl, 1.2 mmol), EDC (191 mg, 1 mmol), and HOBt (41 mg, 304.8 µmol) were added consecutively. After 15 min, compound **3** (433 mg, 1 mmol) was added and the reaction was stirred overnight. Then DCM (40 ml) was added and the solution was washed with water (10 ml). The organic layer was dried with Na_2SO_4, filtered, and concentrated at high vacuum. Finally, the crude product was purified by column chromatography (DCM/MeOH, 96:4) to yield a colorless oil (96.6 mg, 18.3%). ^{1}H NMR (MeOD, 300 MHz): δ 1.50 (s, 54H); 1.55 (s, 27H); 1.71 (m, 18H); 2.42 (t, J = 6 Hz, 6H); 2.49 (m, 2H); 3.20 (t, J = 6 Hz, 6 H); 3.31 (m, 12H);

3.55-3.74 (m, 17H); 6.84 (s, 2H). ESI-MS (m/z): calcd. for (M+Na): 1749.13, found 1748.72; calcd. for (M+2Na): 886.06, found 886.27; calcd. for (M+3Na): 598.37, found 598.52. The tert-butyl groups were then deprotected using a 1:1 (v/v) mixture of TFA: DCM for 2.5 h. After the solvents were removed, the excess of TFA was co-evaporated 4 times with DCM and then the product was dried under high vacuum ^{1}H NMR (MeOD, 300 MHz): δ 1.47 (m, 6H); 1.53 (m, 6H); 1.91 (m, 6H); 2.43 (m, 8H); 3.17 (m, 6H); 3.58-3.65 (m, 15H); 4.1 (m, 14H); 6.82 (s, 2H). ^{13}C NMR ($(CD_3)_2SO$, 125 MHz): δ 23.2, 28.9, 29.0, 33.9, 34.3, 35.9, 38.4, 53.4, 59.7, 64.5, 67.4, 68.2, 134.5, 157.9, 158.2, 158.5, 158.8, 170.0, 170.7, 172.7, and 173.5. HRMS-ESI^{+} (m/z) calcd. for $[M+K]^{+}$, 1259.4457 found, 1259.4465.

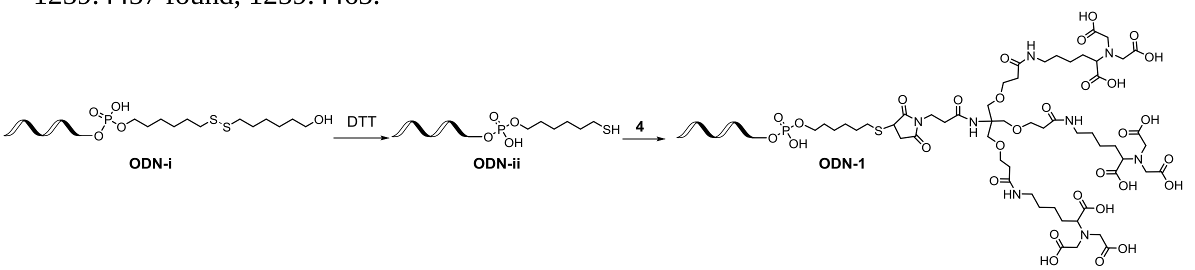

General procedure for the synthesis of the ODN-1 strands: ODN-i (200 nmol) was treated with 400 µl of a DTT solution (50 mM DTT in 50 mM Tris buffer, pH 8.3) for 1 hour. The reduced oligonucleotide (ODN-ii) was then desalted on a Sephadex™ G-25 column and dried under reduced pressure. ODN-ii was added to a solution of **4** (8 mg) in concentrated PBS ×10, pH 7. The reaction was stirred overnight. The product was purified using RP-HPLC. MALDI-TOF MS (m/z): X-ODN-1: calcd. 6319.6, found 6334.2; ODN-1: calcd. 8876.1, found 8893.3; TAMRA-ODN-1: calcd. 11453.6, found 11454.3; ODN-1b: calcd. 9139.8, found 9139.2; ODN-1c: calcd. 9119.9, found 9115.9.

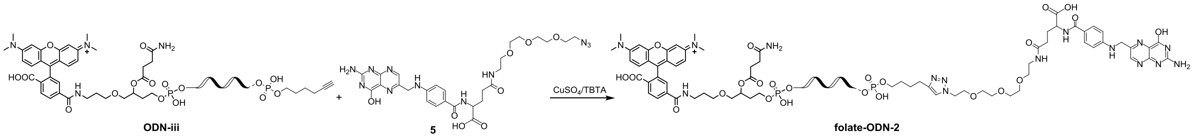

Synthesis of folate-ODN-2: Folate azide **5** was prepared according to a previously published procedure.[3] ODN-iii (150 nmol) was dissolved in 160 µl MQ water, followed by the addition of and the mixture was stirred for 12 h. The product was purified using RP-HPLC to afford folate-ODN-2. MALDI-TOF MS (m/z): calcd. 9940, found 9941.

Strand structures

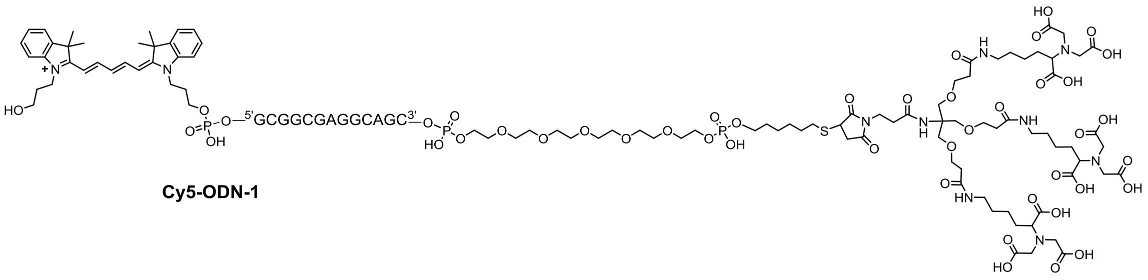

Cy5-ODN-1

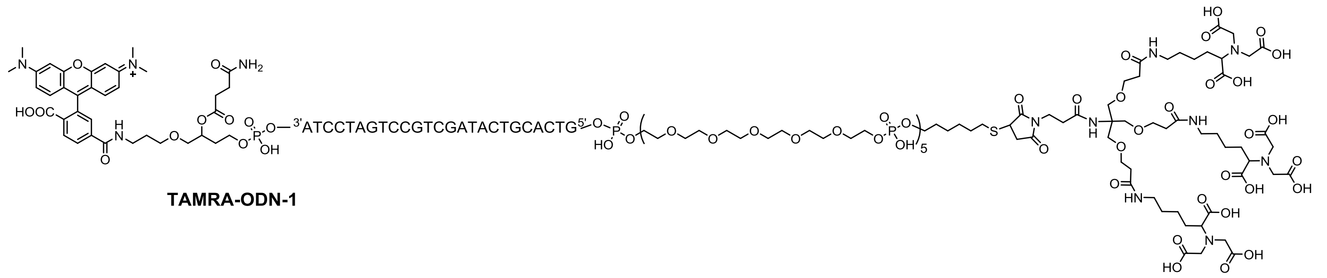

TAMRA-ODN-1

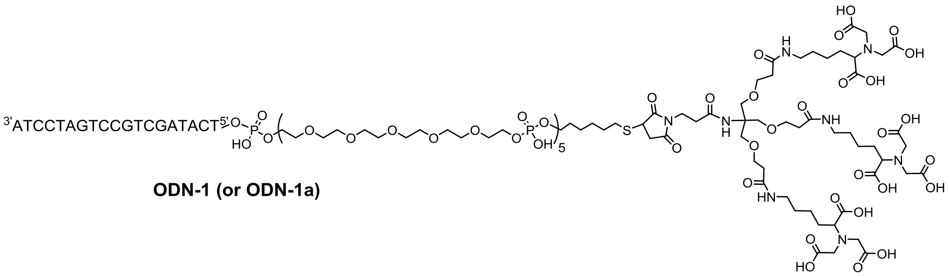

ODN-1 (or ODN-1a)

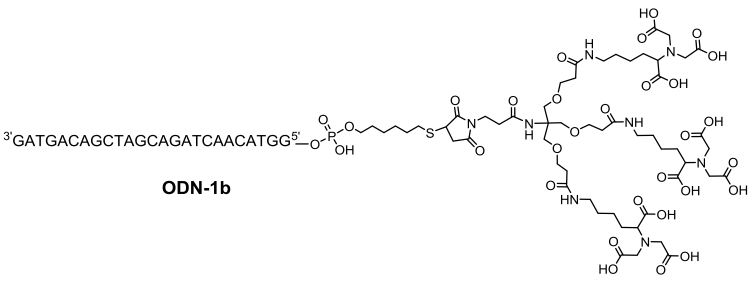

ODN-1b

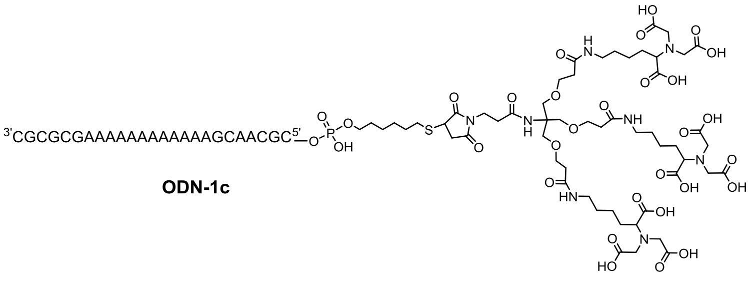

ODN-1c

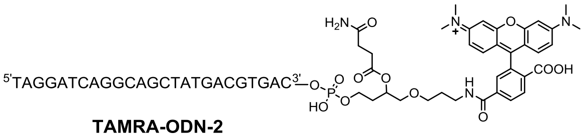

TAMRA-ODN-2

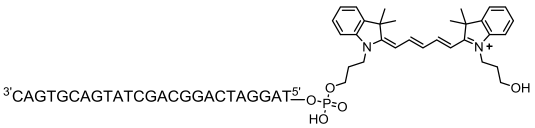

Cy5-ODN-2

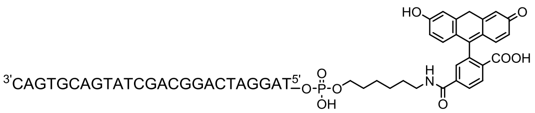

ODN-2a (or FAM-ODN-2)

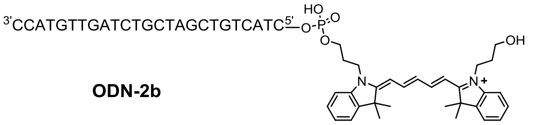

ODN-2b

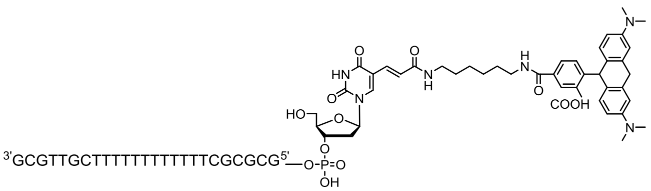

ODN-2c

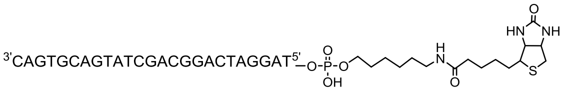

biotin-ODN-2

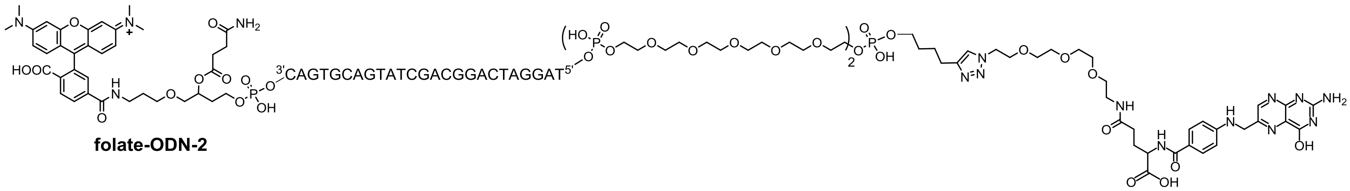

folate-ODN-2

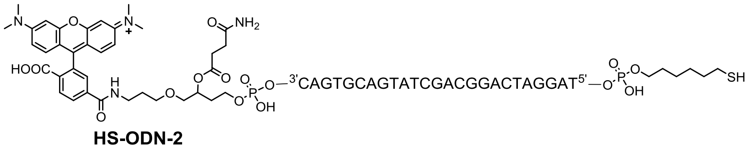

HS-ODN-2

5'GTCACGTCATAGCTGCCTGATCCTA3'

ODN-3

3. OmpC construction and expression

OmpC construction. The *E. coli* outer membrane protein C (OmpC) was isolated by PCR, amplified from the *E. coli* ASKA library[4], and cloned into pET21 using RF cloning[5] (OmpC_FpET21: TTTGTTTAACTTTAAGAAGGAGATATACATATGAAAGTTAAAGTACTGTCCCTC and (OmpC_RpET21:TTCCTTTCGGGCTTTGTTAGCAGCCGGATCTTAGAACTGGTAAACCAGACCC). 3 copies of polyhistidine-linker sequences $(SAGHHHHHGT)_3$ were inserted by in the predicted 7th loop of the OmpC[6] by 3 rounds of consecutive cloning using inverse PCR.[7] The primers that were used to introduce 1 to 3 copies of the polyhistidine linker sequences are: (OMPC_His1F: CATCATCACCATGGTACCTCTAAAGGTAAAAACCTGGGTCGTGGCTAC

OMPC_His1R: ATGGTGATGATGATGATGACCCGCGGAGGTACCATGGTGATGATGGTGATGACCCGCGGA.

OMPC_His2F: CACCATCACGGTACCTCTAAAGGTAAAAACCTGGGTCGTG and OMPC_His2R: GTGATGGTGACCCGCGGAGGTACCATGGTGATGATGGTGATG. OMPC_His3F: CATCATCATGGTACCTCTAAAGGTAAAAACCTGGGTCGTG and OMPC_His3R: ATGATGATGACCCGCGGAGGTACCGTGATGGTGGTGATGGTG).

Purification of OmpC. The expression of OmpC was tested in the whole cell extracts (WCE) and in the membrane fraction. Cultures expressing OmpC and His-OmpC were harvested, resuspended in Na_2HPO_4 (10 mM, pH 7.3), and lyzed by sonication. A sample from each culture was analyzed by SDS-PAGE for the expression of OmpC in the WCE. Following sonication, the supernatant was separated by centrifugation at 13,800g for 10 min. The membrane fraction was recovered by centrifugation of the supernatant at 13,800g for 30 min, resuspended in 10 mM Na_2HPO_4, pH 7.3, 2% Triton X-100, and incubated at 37 °C for 30 min. The insoluble fraction was recovered by centrifugation at 13,800g for 30 min, washed, and resuspended in 10 mM Na_2HPO4, pH 7.3.[8] Proteins from the membrane fractions were analyzed by SDS-PAGE.

4. Supplementary figures

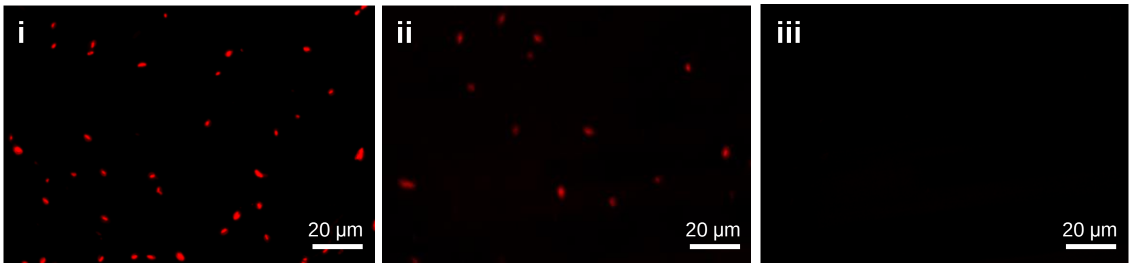

Supplementary Fig. 1. Fluorescence images of (i) bacteria decorated with Cy5-ODN-1 monitored after treatment with benzonase nuclease for (ii) 0.5 h and (iii) 1.5 h.

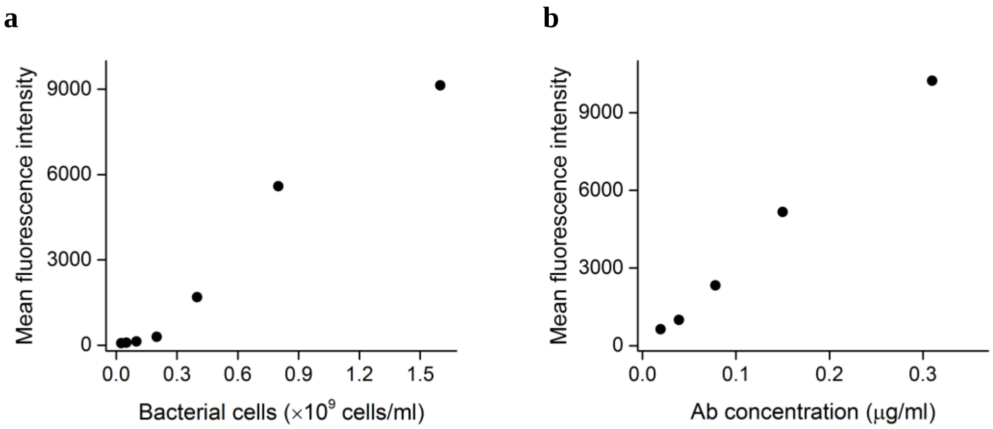

Supplementary Fig. 2. Flow cytometry analysis of KB cells incubated with increasing concentrations of **a** D2-bacteria or **b** anti-FR-Ab.

5. Representative HPLC chromatograms

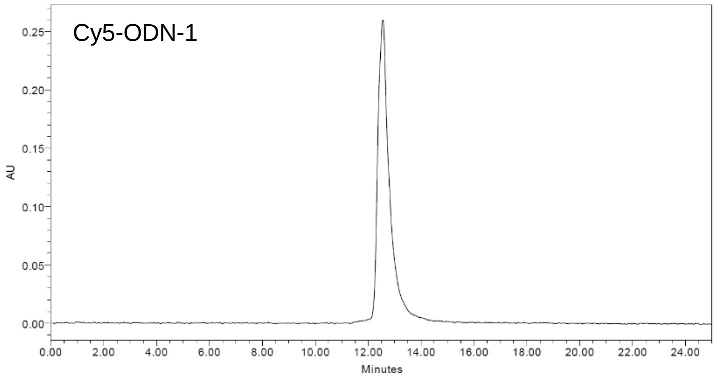

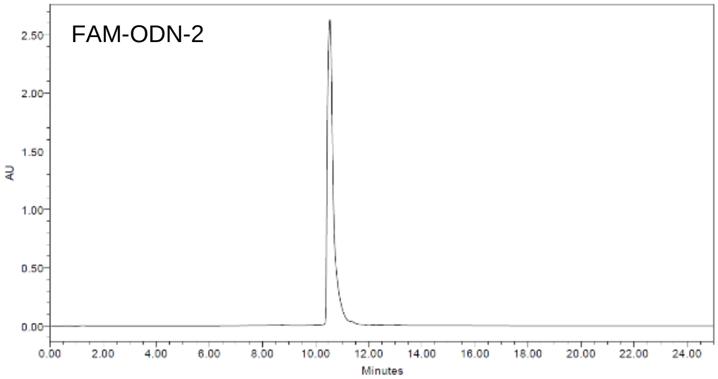

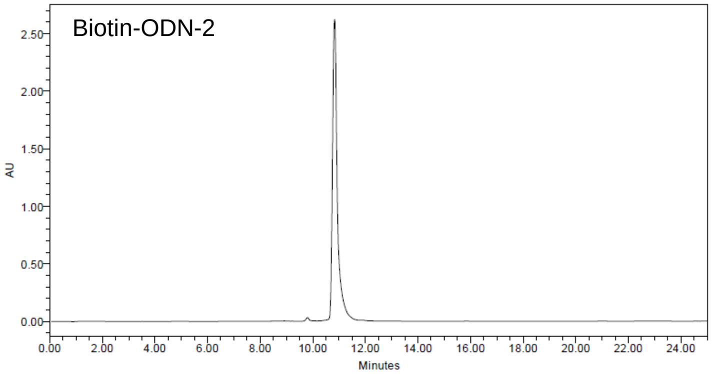

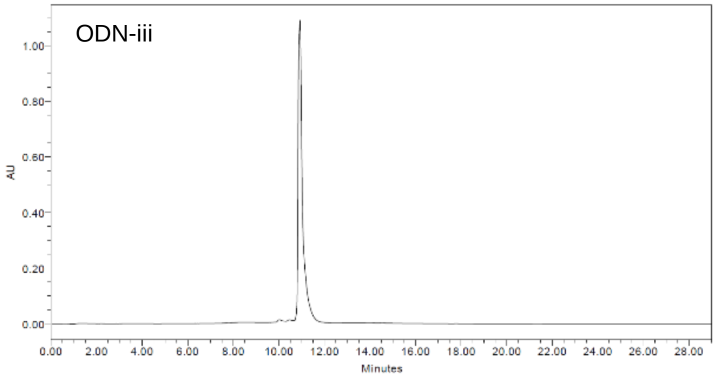

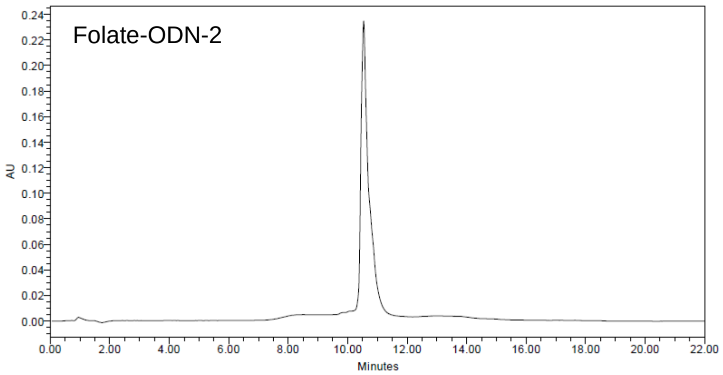

6. References

1. Cardona, C.M. & Gawley, R.E. An improved synthesis of a trifurcated newkome-type monomer and orthogonally protected two-generation dendrons. *J. Org. Chem.* **67**, 1411-1413 (2002).
2. Huang, Z., Park, J.I., Watson, D.S., Hwang, P. & Szoka, F.C. Facile synthesis of multivalent nitrilotriacetic acid (nta) and nta conjugates for analytical and drug delivery applications. *Bioconjugate Chem.* **17**, 1592-1600 (2006).
3. Willibald, J., Harder, J., Sparrer, K., Conzelmann, K.-K. & Carell, T. Click-modified anandamide sirna enables delivery and gene silencing in neuronal and immune cells. *J. Am. Chem. Soc.* **134**, 12330-12333 (2012).
4. Kitagawa, M. et al. Complete set of orf clones of escherichia coli aska library (a complete s et of e. Coli k -12 orf a rchive): Unique resources for biological research. *DNA Res.* **12**, 291-299 (2005).
5. Unger, T., Jacobovitch, Y., Dantes, A., Bernheim, R. & Peleg, Y. Applications of the restriction free (rf) cloning procedure for molecular manipulations and protein expression. *J. Struct. Biol.* **172**, 34-44 (2010).
6. Xu, Z. & Lee, S.Y. Display of polyhistidine peptides on theescherichia colicell surface by using outer membrane protein cas an anchoring motif. *Appl. Environ. Microbiol.* **65**, 5142-5147 (1999).
7. Hemsley, A., Arnheim, N., Toney, M.D., Cortopassi, G. & Galas, D.J. A simple method for site-directed mutagenesis using the polymerase chain reaction. *Nucleic Acids Res.* **17**, 6545-6551 (1989).
8. Puente, J., Juárez, D., Bobadilla, M., Arias, C.F. & Calva, E. The salmonella ompc gene: Structure and use as a carrier for heterologous sequences. *Gene* **156**, 1-9 (1995).
