# Supplementary material for: Decorating bacteria with self-assembled synthetic receptors
Source: Nat Commun. 2020 Mar 10;11:1299. doi: 10.1038/s41467-020-14336-7 (PMC7064574; doi:10.1038/s41467-020-14336-7)
Supplement: Supplementary file 2 — Reporting Summary [file 41467_2020_14336_MOESM2_ESM.pdf]

nature research

Corresponding author(s): David Margulies
Leila Motiei

Reporting Summary

Nature Research wishes to improve the reproducibility of the work that we publish. This form provides structure for consistency and transparency in reporting. For further information on Nature Research policies, see Authors & Referees and the Editorial Policy Checklist.

Statistical parameters

When statistical analyses are reported, confirm that the following items are present in the relevant location (e.g. figure legend, table legend, main text, or Methods section).

n/a	Confirmed	
☐	☒	The exact sample size (*n*) for each experimental group/condition, given as a discrete number and unit of measurement
☒	☐	An indication of whether measurements were taken from distinct samples or whether the same sample was measured repeatedly
☒	☐	The statistical test(s) used AND whether they are one- or two-sided<br>*Only common tests should be described solely by name; describe more complex techniques in the Methods section.*
☒	☐	A description of all covariates tested
☒	☐	A description of any assumptions or corrections, such as tests of normality and adjustment for multiple comparisons
☒	☐	A full description of the statistics including central tendency (e.g. means) or other basic estimates (e.g. regression coefficient) AND variation (e.g. standard deviation) or associated estimates of uncertainty (e.g. confidence intervals)
☒	☐	For null hypothesis testing, the test statistic (e.g. *F*, *t*, *r*) with confidence intervals, effect sizes, degrees of freedom and *P* value noted<br>*Give P values as exact values whenever suitable.*
☒	☐	For Bayesian analysis, information on the choice of priors and Markov chain Monte Carlo settings
☒	☐	For hierarchical and complex designs, identification of the appropriate level for tests and full reporting of outcomes
☒	☐	Estimates of effect sizes (e.g. Cohen's *d*, Pearson's *r*), indicating how they were calculated
☐	☒	Clearly defined error bars<br>*State explicitly what error bars represent (e.g. SD, SE, CI)*

Our web collection on statistics for biologists may be useful.

Software and code

Policy information about availability of computer code

Data collection	Fluorescence images: ImageJ software, Flow cytometer: see below, HPLC: Agilent Chemstation
Data analysis	OriginPro 9.1

For manuscripts utilizing custom algorithms or software that are central to the research but not yet described in published literature, software must be made available to editors/reviewers upon request. We strongly encourage code deposition in a community repository (e.g. GitHub). See the Nature Research guidelines for submitting code & software for further information.

Data

Policy information about availability of data

All manuscripts must include a data availability statement. This statement should provide the following information, where applicable:
- Accession codes, unique identifiers, or web links for publicly available datasets
- A list of figures that have associated raw data
- A description of any restrictions on data availability

The data supporting this study are available in the Supplementary Information or from the corresponding author upon request.

Field-specific reporting

Please select the best fit for your research. If you are not sure, read the appropriate sections before making your selection.

☒ Life sciences ☐ Behavioural & social sciences ☐ Ecological, evolutionary & environmental sciences

For a reference copy of the document with all sections, see nature.com/authors/policies/ReportingSummary-flat.pdf

Life sciences study design

All studies must disclose on these points even when the disclosure is negative.

Sample size	N/A
Data exclusions	N/A
Replication	All experiments are reproducible.
Randomization	N/A
Blinding	N/A

Reporting for specific materials, systems and methods

Materials & experimental systems

n/a	Involved in the study
☒	☐ Unique biological materials
☐	☒ Antibodies
☐	☒ Eukaryotic cell lines
☒	☐ Palaeontology
☒	☐ Animals and other organisms
☒	☐ Human research participants

Methods

n/a	Involved in the study
☒	☐ ChIP-seq
☐	☒ Flow cytometry
☒	☐ MRI-based neuroimaging

Antibodies

Antibodies used	PE anti-FoR1 antibody (Biolegend, Cat. No. BLG-908303)<br>PE mouse IgG2a, κ isotype (Biolegend, Cat. No. BLG-400213)
Validation	The antibodies were validated by the manufacturer.<br>PE anti-FoR1 antibody : https://www.biolegend.com/en-us/products/pe-anti-folr1-folate-binding-protein-antibody-13690<br>PE mouse IgG2a, κ isotype: https://www.biolegend.com/en-us/products/pe-mouse-igg2a--kappa-isotype-ctrl-fc-3043

Eukaryotic cell lines

Policy information about cell lines

Cell line source(s)	KB cells and MCF-10A
Authentication	The cell lines were not authenticated.
Mycoplasma contamination	The cell lines were screened negative for mycoplasma using a PCR-based assay (EZ-PCR mycoplasma detection kit, Biological Industries).
Commonly misidentified lines (See ICLAC register)	N/A

Flow Cytometry

Plots

Confirm that:

- [x] The axis labels state the marker and fluorochrome used (e.g. CD4-FITC).
- [x] The axis scales are clearly visible. Include numbers along axes only for bottom left plot of group (a 'group' is an analysis of identical markers).
- [x] All plots are contour plots with outliers or pseudocolor plots.
- [x] A numerical value for number of cells or percentage (with statistics) is provided.

Methodology

Sample preparation	Sample preparation is described in the method section of the manuscript.
Instrument	BD FACSAria Fusion instrument (BD Immunocytometry Systems)
Software	BD FACS Diva software v8.0.1 (BD Biosciences), FlowJo software v10.2 (Tree Star)
Cell population abundance	Cell sorting process is not performed.
Gating strategy	FSC-A and SSC-A detectors sensitivities were set to measure KB cells (gating out background of debris and aggregates) via linear scale. FSC-W vs FSC-H and SSC-W vs SSC-H were used to gate out doublets.<br>DAPI was detected by excitation at 405 nm and collection of emission using a 450/50 bandpass (BP) filter. DAPI-A was used to gate only on live cells, via log scale.<br><br>FSC-H and SSC-H detectors sensitivities were set to measure bacteria cells (gating out background of debris and aggregates) via log scale. The corresponding bacteria population was gated for further according to FITC-A, Cy5-A and TAMRA-A fluorescence via log scale as illustrated below and in the manuscript.<br>FITC was detected by excitation at 488 nm and collection of emission using 502 longpass (LP) and 530/30 bandpass (BP) filters. Cy5 was detected by excitation at 640 nm and collection of emission using a 670/30 BP filter. TAMARA was detected by excitation at 561 nm and collection of emission using a 582/15 BP filter.<br>Appropriate detectors sensitivities were set via log scale.

- [ ] Tick this box to confirm that a figure exemplifying the gating strategy is provided in the Supplementary Information.
